# Standards of evidence in chronobiology: critical review of a report that restoration of *Bmal1 *expression in the dorsomedial hypothalamus is sufficient to restore circadian food anticipatory rhythms in *Bmal1*-/- mice

**DOI:** 10.1186/1740-3391-7-3

**Published:** 2009-03-26

**Authors:** Ralph E Mistlberger, Ruud M Buijs, Etienne Challet, Carolina Escobar, Glenn J Landry, Andries Kalsbeek, Paul Pevet, Shigenobu Shibata

**Affiliations:** 1 Department of Psychology, Simon Fraser University, Burnaby, BC Canada; 2 Instituto de Investigacíones Biomedicas, Universidad Nacional Autónoma de México, Mexico; 3 Institut de Neurosciences Cellulaires et Intégratives, UPR3212, Centre National de la Recherche Scientifique, Université de Strasbourg, Strasbourg, France; 4 Departamento de Anatomía, Fac de Medicina, Universidad Nacional Autónoma de México, Mexico; 5 Netherlands Institute for Neuroscience, Amsterdam, The Netherlands; 6 Department of Pharmacology, School of Science and Engineering, Waseda University, Tokyo, Japan

## Abstract

Daily feeding schedules generate food anticipatory rhythms of behavior and physiology that exhibit canonical properties of circadian clock control. The molecular mechanisms and location of food-entrainable circadian oscillators hypothesized to control food anticipatory rhythms are unknown. In 2008, Fuller et al reported that food-entrainable circadian rhythms are absent in mice bearing a null mutation of the circadian clock gene *Bmal1 *and that these rhythms can be rescued by virally-mediated restoration of *Bmal1 *expression in the dorsomedial nucleus of the hypothalamus (DMH) but not in the suprachiasmatic nucleus (site of the master light-entrainable circadian pacemaker). These results, taken together with controversial DMH lesion results published by the same laboratory, appear to establish the DMH as the site of a *Bmal1*-dependent circadian mechanism necessary and sufficient for food anticipatory rhythms. However, careful examination of the manuscript reveals numerous weaknesses in the evidence as presented. These problems are grouped as follows and elaborated in detail: 1. data management issues (apparent misalignments of plotted data), 2. failure of evidence to support the major conclusions, and 3. missing data and methodological details. The Fuller et al results are therefore considered inconclusive, and fail to clarify the role of either the DMH or *Bmal1 *in the expression of food-entrainable circadian rhythms in rodents.

## Review

Circadian rhythms in mammals are regulated by a master circadian pacemaker located in the suprachiasmatic nucleus (SCN) [1,2]. This pacemaker mediates entrainment of circadian rhythms to daily light-dark (LD) cycles, but is not necessary for entrainment of circadian rhythms to daily feeding schedules [3-5]. Rats, mice and other species entrained to a LD cycle and restricted to a single meal (typically 2–4 h duration) at a fixed time each day exhibit increased locomotor activity beginning 1–3 h prior to mealtime. Once established (typically within a week of scheduled feeding) this daily rhythm of food anticipatory activity persists when meals are omitted for 2 or more days (i.e., activity remains concentrated near the expected mealtime). Food-anticipatory rhythms are most readily generated by feeding schedules with a stable periodicity in the circadian range (~22–31-h), and are not affected by complete ablation of the SCN. Therefore, a food-sensitive circadian timing mechanism regulating behavior must exist in the brain or periphery outside of the SCN [6-8]. This mechanism has been conceptualized as a food-entrainable oscillator or pacemaker, analogous to the light-entrainable pacemaker in the SCN.

Efforts to localize food-entrainable circadian oscillators for behavior began over 30 years ago, but until the turn of the 21^st ^century were limited to a few laboratories, and yielded primarily negative findings. With the advent of powerful new molecular biological techniques, and recognition of the importance of food as a time-cue for circadian oscillators outside of the SCN [9,10], more laboratories have undertaken work on the neurobiology of food-entrainment. One laboratory (Saper and colleagues) has now published two studies that, taken together, appear to have succeeded in localizing a brain site critical for the expression of food anticipatory rhythms. In the first study, Gooley et al [11] reported that ablation of ~70–90% of the dorsomedial hypothalamus (DMH), by localized injection of the neurotoxin ibotenic acid, severely attenuated or eliminated food anticipatory rhythms of activity, sleep-wake and temperature rhythms in rats. In the second study, Fuller et al [12] exploited gene knockout and rescue technology to show that food-anticipatory activity and temperature rhythms are absent in mice lacking the circadian clock gene *Bmal1*, and are rescued by virally-mediated restoration of *Bmal1 *expression selectively in the DMH (but not in the SCN). The two studies appear to establish that the DMH contains *Bmal1*-dependent circadian oscillators that are both necessary and sufficient for the expression of food-entrainable behavioral and temperature rhythms in rodents.

These studies potentially constitute a seminal demonstration of localization of function in the mammalian brain. However, despite considerable effort, other laboratories, using rats or mice, have so far been unable to confirm either the lesion or the gene knockout results [13-18]. Consequently, the two studies demand close scrutiny. Commentaries on Gooley et al [11] are already available [19,20]. Here we present a comprehensive analysis of the Fuller et al [12] study, with major points of concern grouped and numbered for clarity. In all references to figures in the Fuller et al text, the figure number is spelled out (e.g., Figure one). Supplementary figures in Fuller et al will be identified by the letter 'S'.

### 1. Data management issues

A critical task for peer reviewers is to evaluate whether the evidence presented in a new study supports the authors' substantive conclusions. Before undertaking this task, the reviewers must have confidence that the evidence has been presented both fully and accurately. Accuracy is generally assumed, unless there are clear indications (e.g., internal inconsistencies) that errors may have occurred. Inspection of the figures provided in Fuller et al [12] suggests that there may be significant errors in the alignment and labeling of data displays critical to evaluating the claims of the study.

**Figure 1:**
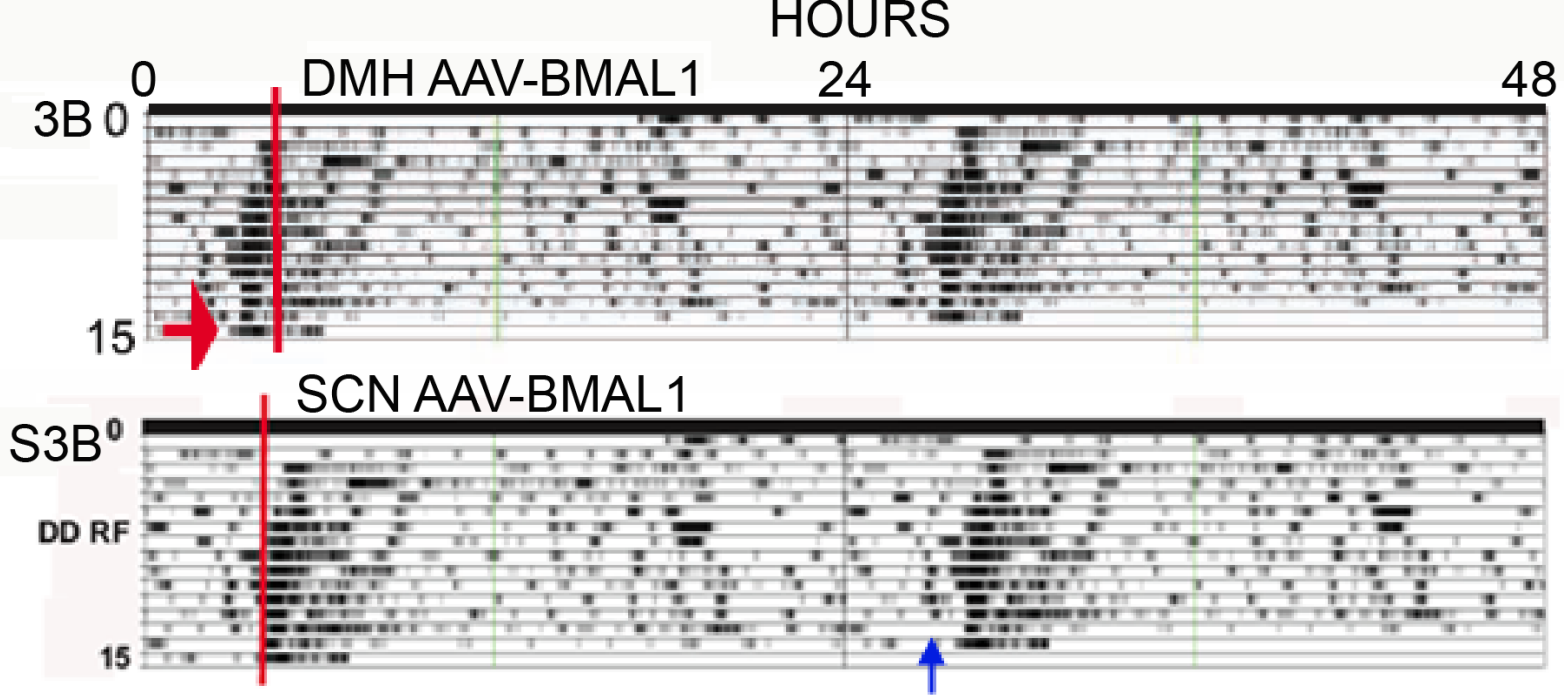
**Duplicate 'actogram style' charts (modified, with permission, from Fuller et al **[12]**^© ^(2008) AAAS , Figures 3B and S3B, original supplementary online materials)**. The blue arrow indicates the ~3-h section that differs between the two versions of these data.

1a. The Fuller et al [12] paper was published with three multi-panel figures in the main text, and four supplementary figures available on-line. Figure three B in the main text is an 'actogram style' double-plot of core body temperature data intended to illustrate recovery of food anticipation in a *Bmal1*-/- mouse by adeno-associated viral (AAV)-BMAL1 injection into the DMH. Figure S3B in the original supplementary materials was another double-plot of body temperature intended to illustrate failure of recovery of food anticipation following injection of AAV-BMAL1 into the SCN. However, the two double-plots (Fig. 1 here) were clearly the same data, differing by ~3 h in the start-time, and in the placement of a red line intended to denote the onset of daily mealtime. Notably, the two charts appear to be identical except for an ~3 h segment just prior to mealtime on the second to last day of restricted feeding (Fig. 1; see the blue arrow in panel S3B). Five months after publication (Science, Oct. 31, 2008), the duplicate double-plot in the on-line supplementary materials was replaced by another plot, accompanied by the following Correction: "*Figs. S2 and S3 have been replaced. In Fig. S2, panels B and C were reversed; the legend for panel B described panel C, and the legend for panel C described panel B. In addition, Fig. S3B contained an error, a result of mistakenly using an incorrect file to make the plot. The incorrect file was an incomplete working file obtained from the same animal and experiment as shown in *Fig. 3B*in the main text, but with an incorrect start time (which advanced the phase). Fig. S3D, in which the trace is derived from the data shown in fig. S3B, was also incorrect*." The unidentified 'error' presumably refers to the mismatch between the two figures. It is not clear how the duplicate plots could appear to be identical except for one critical segment immediately preceding mealtime. This could be a peculiarity of the algorithms used to generate the 'actogram' style plots, but neither the original paper nor the supplementary materials provide information on the plotting conventions of this software. Regardless, the fact that a significant misalignment occurred raises concerns about the accuracy of other figures in the paper.

**Figure 2:**
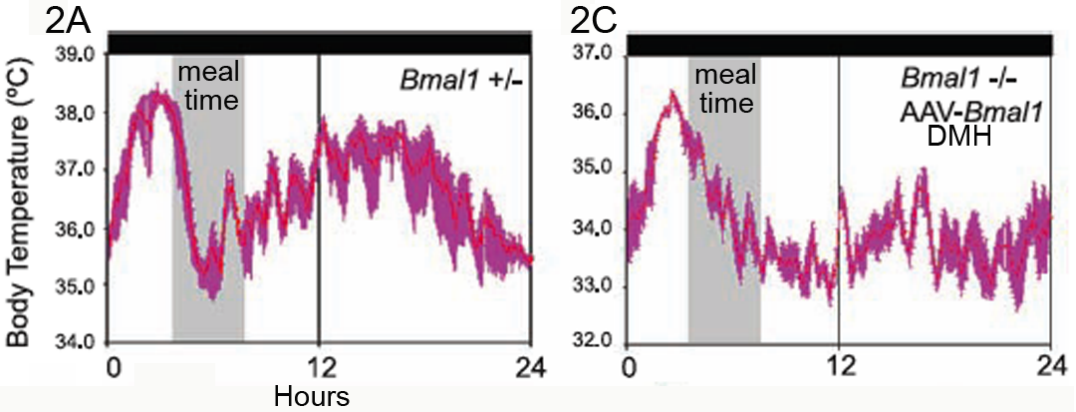
**Average waveforms of body temperature in food restricted mice (modified, with permission, from Fuller et al **[12]**^© ^(2008) AAAS , Figure S3C)**. In both waveforms, temperature peaks prior to mealtime and begins dropping before mealtime, with no evidence of feeding induced thermogenesis. See also Fig. 3C (adapted from Fuller et al Figure S3C).

**Figure 3:**
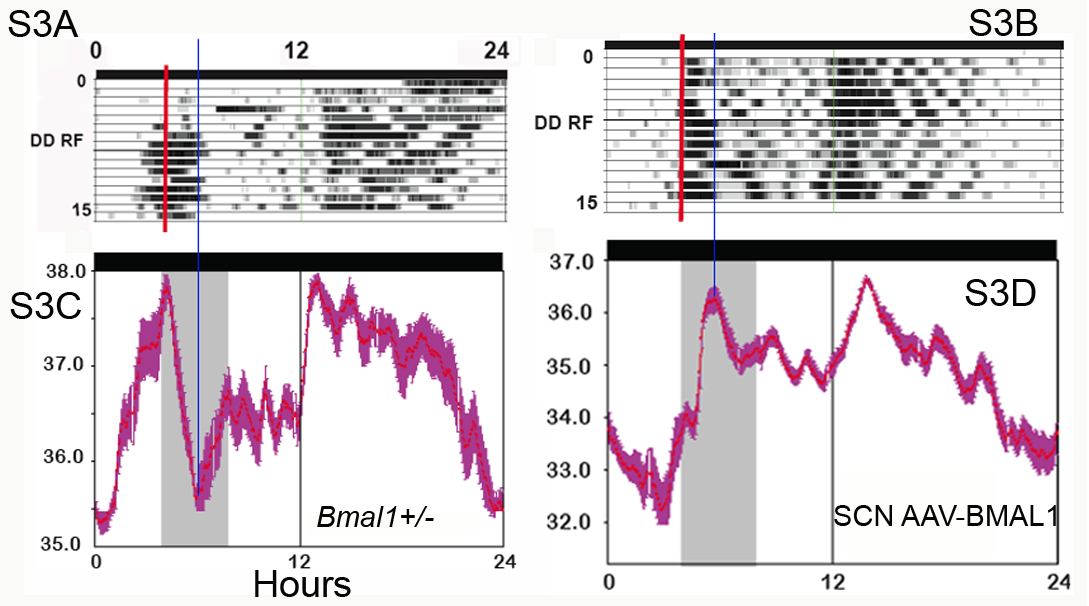
**Actogram-style plots and corresponding average waveforms of body temperature in food restricted mice (modified, with permission, from Fuller et al **[12]**^© ^(2008) AAAS , Figure S3)**. For clarity, we have placed the waveform figures under the corresponding actogram-style figures. According to the text, these 2 waveforms were derived from days 10–14 of restricted feeding. We have aligned the waveforms and corresponding actogram-style plots, and drawn a blue line through the trough of body temperature that occurred (without explanation) in the middle of mealtime in waveform C, and through the peak in temperature that occurred in the middle of mealtime in waveform D. Clearly, the peak in temperature in D is not reflected by the density of the same data in B. In addition, the temperature curve in both waveforms is a mirror image on either side of the blue line. Therefore, in the actogram-style plots of the same data, the dark sections (indicating higher temperature) should also be symmetrical on either side of the blue line. They are not. In actogram-style plot B, high temperature is indicated during the first 1–2 h of mealtime, yet the corresponding waveform shows a lower temperature (likely below the daily mean) during that time.

**Figure 4:**
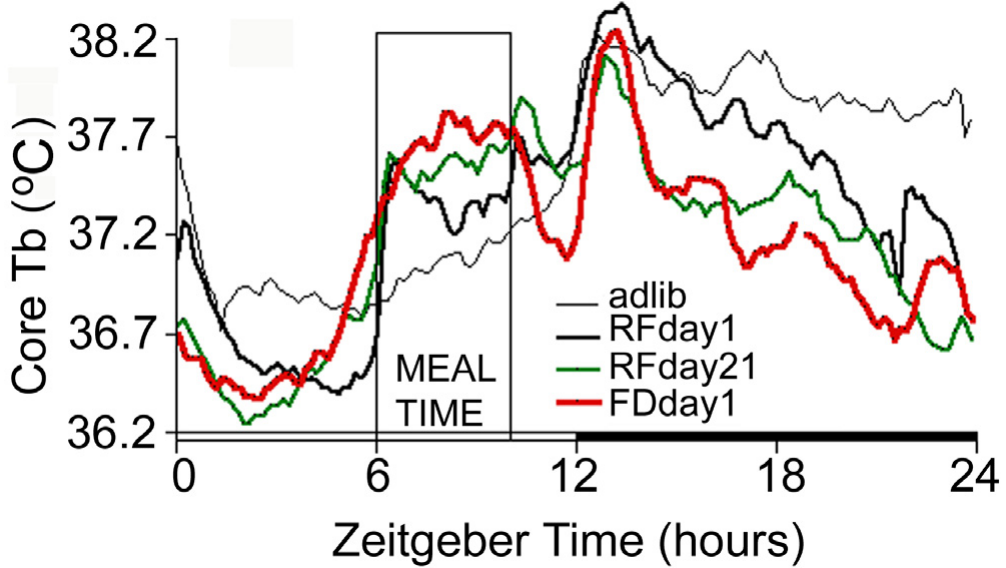
**The thermogenic effect of midday feeding in rats (R. Mistlberger, B. Kent, G. Landry, unpublished)**. Group mean average waveforms of core body temperature measured via implanted transponders in rats (N = 11) under adlib food access (thin black line), 4 h/day restricted feeding (heavy line = day1, heavy green line = day 21), and total food deprivation (heavy red line = day1). Temperature rises dramatically within 10 min of meal onset on days 1 and 21 of restricted feeding, and remains elevated throughout mealtime on the meal omission day after day 21.

**Figure 5:**
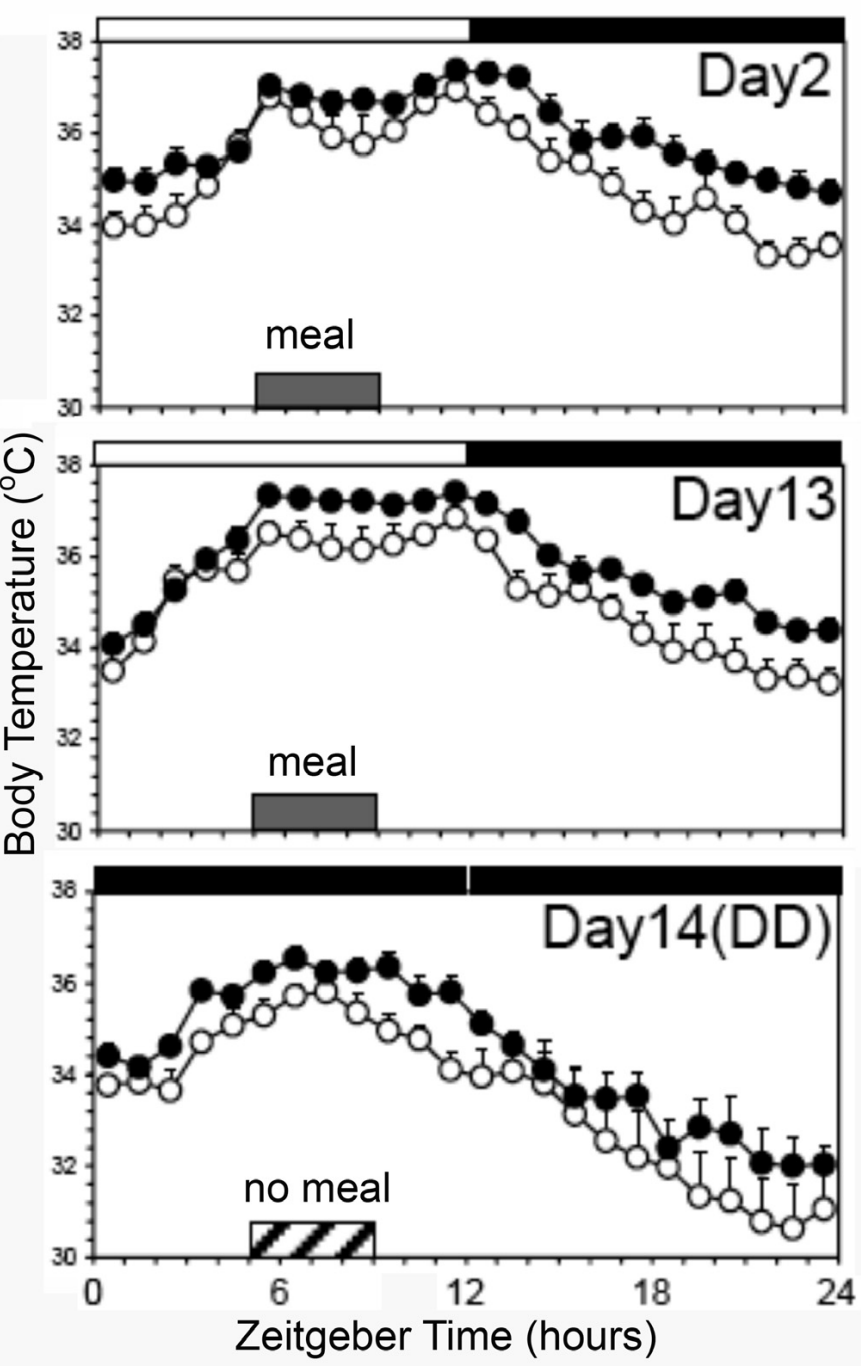
**The thermogenic effect of midday feeding in mice, adapted from Moriya et al **[16]. Group mean average waveforms of core body temperature measured via implanted transponders in mice (sham lesion = open circles, DMH lesion = closed circles) under 4 h/day restricted feeding in LD (days 2 and 13) and total food deprivation in DD. Temperature rises dramatically within 15 min of meal onset on days 2 and 13 of restricted feeding, and remains elevated throughout mealtime on the meal omission day.

1b. Careful examination of the other 'actograms' and average waveforms in Fuller et al suggest that other alignment errors may well have occurred. One striking indicator of possible errors is the unusual direction and rapidity of change of body temperature in several of the waveforms. According to the figure legends, Figure two and corrected Figure S3 depict waveforms of body temperature averaged over days 10–14 of 4-h/day restricted feeding. Figures two A and C (Fig. 2 here) and S3C (Fig. 3 here) illustrate a food anticipatory rise of body temperature in heterozygous or DMH-rescued AAV-BMAL1 null mutant mice. In Figures two A and C, body temperature peaks about 1–2 h prior to mealtime, and then drops monotonically during mealtime. In Figure S3C, temperature peaks at the onset of mealtime, and then falls precipitously during mealtime. These waveforms appear to violate a fundamental metabolic consequence of food intake. When small rodents eat, there is a significant rise of brain and body temperature, reflecting a thermogenic effect of food intake and associated activity [21]. This is normally readily apparent in measures of temperature from brain, muscles and the intraperitoneal cavity, and represents a physiological signature of mealtime (for examples from rats and mice recorded in other laboratories, see Figs. 4 and 5 here and [22]). The absence of this thermogenic effect of food intake in Fuller et al could mean any of the following: 1. the mice may not have been fed on these days (unlikely, given that locomotor activity, and therefore temperature, if high prior to mealtime, normally remain elevated at least through the expected mealtime), 2. the data may be misaligned, and the waveforms shifted to the left or the right of where they should be relative to mealtime, or 3. rather than body temperature, the data may actually be locomotor activity, which typically does decrease rapidly while rats and mice eat for an hour or so and then take a post-prandial pause before eating again. Errors of these types are not mutually exclusive (e.g., some waveforms appear misaligned, and others exhibit characteristics of activity data rather than temperature data). These inconsistencies raise questions about the reliability of the data analysis procedures used in the study.

1c. Data misalignment in Fuller et al is also indicated by mismatches between waveforms and actogram-style plots. In Figure S3 (the Corrected version on-line) the waveforms depicted in panels three C and D (Fig. 3C, D here) are said to have been derived by averaging the last 4 days of food restriction depicted in the corresponding actogram-style temperature plots (lines 10–14 on panels three A and B, respectively). However, neither temperature waveform matches its temperature actogram-style plot. The actogram-style plot in panel A clearly shows a period of high temperature for at least the first 2 h (and probably 3 h) of mealtime, followed by low temperature. The derived average waveform (panel C, below it) shows a rapid drop of temperature over the first 2 h, and then a rise to half maximal by hour 4 of mealtime. The actogram-style temperature plot in panel B shows high temperature during hours 1–2 of mealtime, and then lower temperature during hours 3–4, yet the derived average waveform shows temperature that is much lower during the first 1–1.5 h of mealtime, and then rises to maximal values during the middle of mealtime, with sustained high levels until after mealtime. The average waveforms and actogram-style plots exhibit clear discrepancies that could be caused if one is misaligned relative to the other. This misalignment issue exists regardless of whether these are temperature data or are activity data mistakenly labeled in units of temperature (a serious concern for Figure 3A and 3C).

1d. The data illustrated in Figure S3 (Fig. 3 here) have additional characteristics indicating possible mislabeling. Panels S3A and S3C are identified in the supporting online materials as temperature data from a heterozygous mouse on restricted food access in DD. This mouse exhibits a robust 'subjective night' of ~10–12 h duration, recurring every day beginning ~5 h after mealtime. This precise 24-h rhythm is presumably driven by the SCN (the authors comment on the absence of a *'circadian rise in body temperature during the presumptive dark cycle, CT12-24'*, in reference to Figure two C in the main text). However, the published literature on food-restricted mice in DD (e.g., [22-25]) indicates that the SCN-driven rhythm either free-runs without entraining to mealtime, or entrains to mealtime with a positive phase angle. Fuller et al [12] fail to comment on the peculiarity of their data, relative to results of other mouse studies. A similar extended subjective night is evident in the data from the SCN-rescued AAV-BMAL1 null mutant mouse illustrated in Panels S3B and S3D (Fig. 3 here). The rhythms in these two mice look strikingly similar to the rhythms of wildtype mice entrained to a 24 h LD cycle, with food restricted to the middle of the light period. The authors indicate in the text that in a preliminary experiment, mice were fed at ZT4-8 in LD 12:12. Although the results of that experiment were not reported, the fact that the authors apparently did run a food restriction experiment in LD raises concern that data files were mislabeled and that mice recorded in LD were mistakenly used to represent mice recorded in DD.

### 2. Data inadequate to support major claims

The preceding analysis raises legitimate concerns about data management in Fuller et al [12]. In the next section, we suspend judgment on this issue, and assess whether the data, taken at face value, support the authors' substantive conclusions.

**Figure 6:**
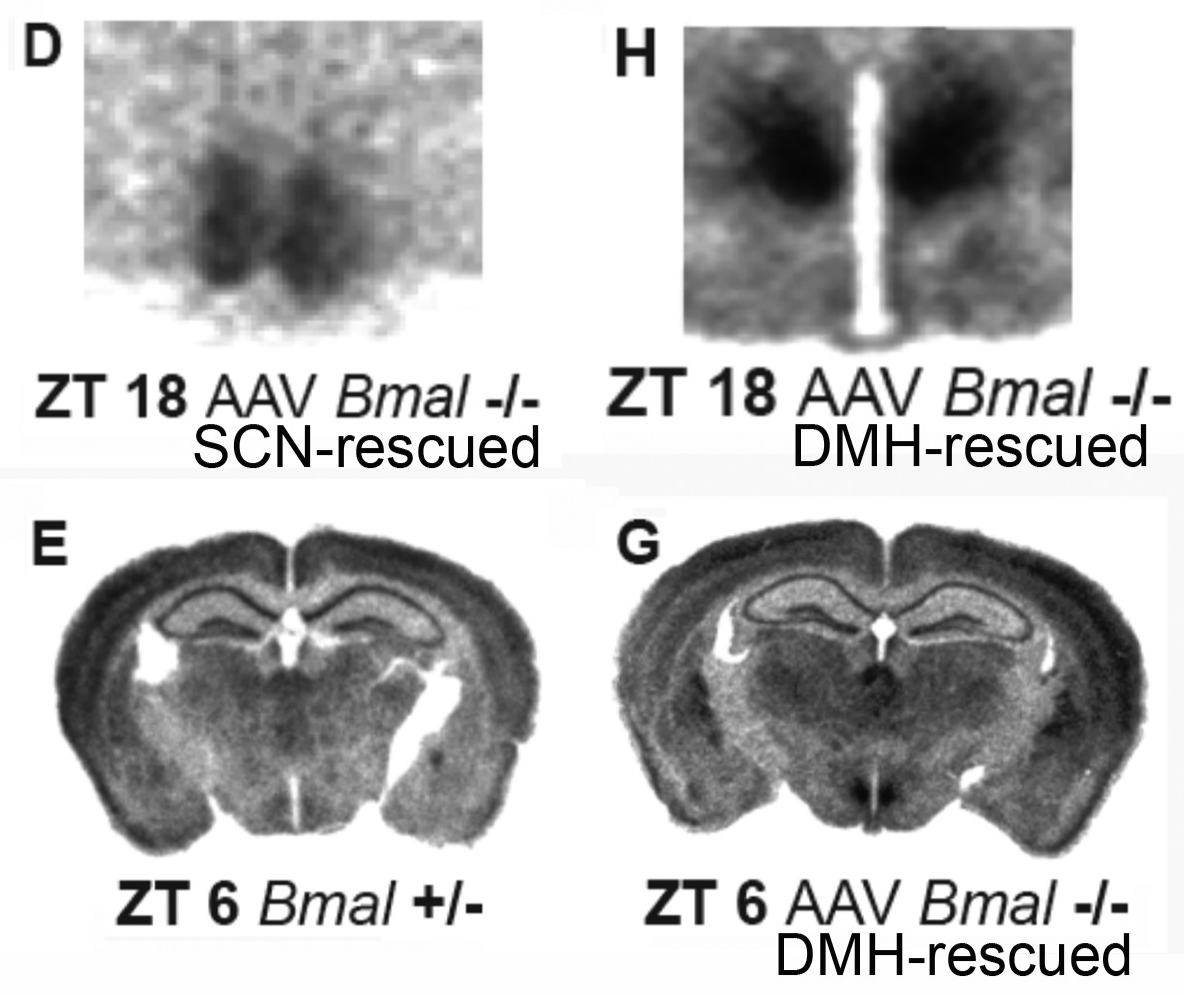
***Bmal1 *and *Per1 *expression in a *Bmal1*+/- control mouse and a *Bmal1*-/- mouse that received intra-DMH AAV-BMAL1 injections bilaterally (modified, with permission, from Fuller et al **[12]**^© ^(2008) AAAS , Figure S4)**. *Bmal1 *expression was restored bilaterally and symmetrically by AAV-BMAL1 injections into the SCN (Panel D) or DMH (panel H) in *Bmal1*-/- mice. The panels do not include other structures that normally express *Bmal1 *in control mice to confirm that *Bmal1 *expression was restricted to the SCN or DMH in null mutants. Panels E and G illustrate *Per1 *expression in full coronal sections from a *Bmal1*+/- control mouse and a *Bmal1*-/- mouse that received an intra-DMH AAV-BMAL1 injection.

2a. One major claim critical to the substantive conclusions of Fuller et al is that injection of AAV-BMAL1 directly into either the SCN or the DMH of *Bmal1*-/- mice selectively restored *Bmal1 *expression in these structures, and selectively rescued light-entrainable and food-entrainable circadian rhythms, respectively (a double dissociation). To demonstrate that restoration of *Bmal1 *expression was indeed spatially restricted to the target structure, it is necessary to provide autoradiographs of *Bmal1 *expression in one or more whole coronal sections of the brain, showing expression in the target structure and no expression outside of this structure in brain regions that normally express *Bmal1 *in wildtype mice at that phase of the DMH rhythm. Figure S4 in Fuller et al shows *Per1 *expression in whole brain coronal sections, but the autoradiographs provided to illustrate *Bmal1 *expression in AAV-BMAL1 mice were cropped to include only the target area (either the SCN or the DMH; Figures one F, two H, S4D, and S4H; see Fig. 6 here). In many studies it is acceptable to present images cropped to focus on a particular target region. However, the claim of the Fuller et al paper is that *Bmal1 *expression in AAV-BMAL1 mice was limited to the SCN or DMH. Consequently, the appropriate standard of evidence is to show selectivity. The critical molecular evidence for regionally selective rescue of *Bmal1 *gene expression is therefore missing from the paper.

Given the authors' claim that *Bmal1 *expression was selectively restored in the DMH, the *Per1 *autoradiographs in Figure S4 (Fig. 6 here) are puzzling. Null mutations of *Bmal1 *result in very low expression of *Per1 *throughout the brain [26]. However, in Figure S4, *Per1 *expression outside of the SCN and DMH is very similar if not identical in the examples provided from *Bmal1*+/-, *Bmal1*-/- and AAV-BMAL1 mice. This similarity is particularly striking by comparing panel S4E and S4G (Fig. 6E, G here), identified as *Bmal1*+/- and AAV-BMAL1 null mutant mice, respectively. In the brain represented by panel G, the viral vector was microinjected into the DMH, but *Per1 *expression is evident in numerous regions outside of the hypothalamus, and looks equivalent to the distribution and intensity of mRNA signal in the heterozygous mouse (panel E), and very different from comparable autoradiographs in the original Bunger et al [26]*Bmal1 *knockout study. The autoradiographs therefore appear at odds with the claim in the text that *Bmal1 *expression in this mouse was limited to the DMH.

**Figure 7:**
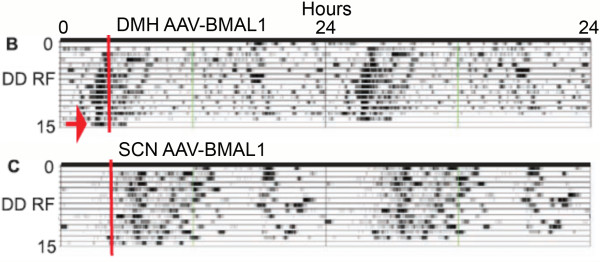
**Actogram-style plots of body temperature during restricted feeding from *Bmal1*-/- mice with or without AAV-BMAL1 injections into the DMH (modified, with permission, from Fuller et al **[12]**^© ^(2008) AAAS , Figure 3)**. Panel B. *Bmal1*-/- mouse that received AAV-BMAL1 injection to DMH. Panel C. *Bmal1*-/- mouse that received no injection. Red line denotes mealtime. Red arrow denotes 24 h food deprivation test.

2b. A second major claim critical to the substantive conclusions of this paper is that food anticipatory rhythms of activity and temperature in *Bmal1*-/- mice were rescued by intra-DMH injection of AAV-BMAL1. However, the 'actogram' style double-plot of body temperature that is provided as evidence of functional rescue (Figure three B; see Figs. 1 and 7 here) illustrates only the food restriction days, and omits any baseline data from ad-lib food access days. On the food restriction days that are shown, temperature rises before mealtime, i.e., it displays a rhythm with an anticipatory phase angle. To interpret these data, it is necessary to see activity and temperature during ad-lib food access PRIOR TO the feeding schedule. Without these baseline data, we do not know whether the rhythms evident during food restriction were already present prior to food-restriction, with a phase that happened by coincidence to be anticipatory to the stated mealtime during food restriction. The critical behavioral evidence for functional rescue of food-entrained rhythms is therefore missing from the paper.

2c. A third claim critical to the substantive conclusions of this paper is that the food anticipatory rhythms rescued in null mutants receiving AAV-BMAL1 in the DMH were 'true' circadian rhythms, because, as stated by the authors, these rhythms persisted "*during a 24-h fast at the end of restricted feeding, demonstrating the circadian nature of the response*" (see panel 3B, Fig. 7 here). However, 24 h does not constitute a test of rhythm persistence. A 24 h food deprivation test is no different from a regular day of food restriction. To establish that a food anticipatory rhythm is the output of a circadian oscillator, and not an hourglass process, food must be removed for at least 2 circadian cycles. This is easily tolerated by rats, but may not be well tolerated by mice, particularly metabolically compromised mutant lines, such as *Bmal1*-/- mice. Nonetheless, the data critical to '*demonstrating the circadian nature' *of food anticipation is the second day of food deprivation, not the first. This study does not include a second day of food deprivation. Therefore, contrary to the authors' claim, this study does not demonstrate the circadian nature of food anticipatory rhythms evident in *Bmal1*-/- mice receiving DMH injections of AAV-BMAL1.

**Figure 8:**
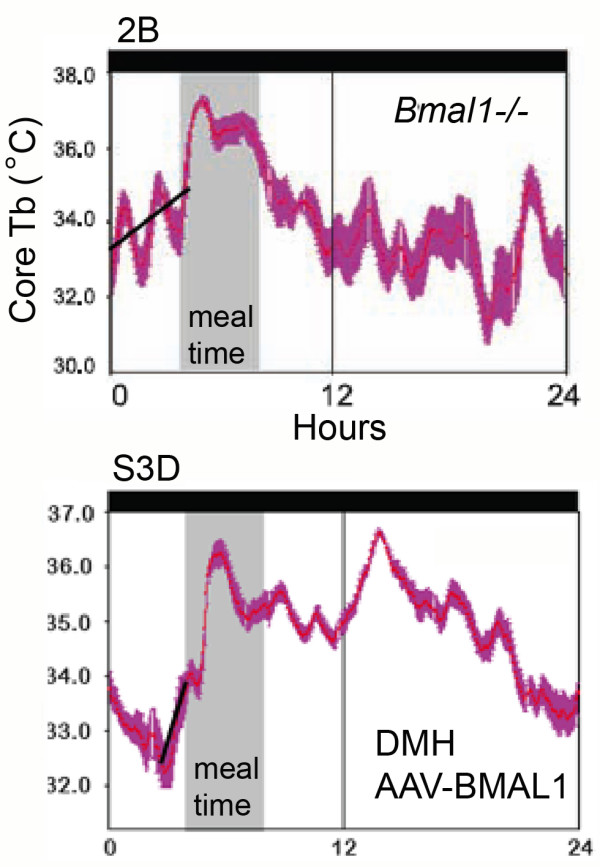
**Body temperature in *Bmal1*-/- mice during restricted daily feeding (modified, with permission, from Fuller et al **[12]**^© ^(2008) AAAS , Figure 2B, left, and S3D, right)**. Neither mouse received AAV-BMAL1 injections. The black regression lines were added here.

2d. A fourth major claim of this paper is that *Bmal1*-/- mice do not exhibit an anticipatory rise of body temperature prior to a 4 h daily meal. To support this claim, data were averaged across restricted feeding days for individual mice and displayed as waveforms. However, inspection of these waveforms reveals that body temperature is in fact clearly rising prior to mealtime in both of the two *Bmal1*-/- mouse examples provided (Fuller et al Figures two C, S3D; Fig. 8 here). The slope of this rise looks less dramatic compared to the two heterozygous mice provided as examples (Fuller et al Figures two A, S3A; Figs. 2 and 4 here), but this is partly because the authors chose to extend the temperature scale across a wider range in the *Bmal1*-/- examples than in the heterozygous examples on the same figures. Thus, in Fuller et al's Figure two A (heterozygote) the temperature scale ranges from 34–38°C degrees, while in Figure two B (null mutant) the temperature scale ranges from 30–38°C. Similarly, in Fuller et al's Figure S3A (heterozygote) the temperature scale ranges from 35–38°C, while in Figure S3D (null mutant) it ranges from 31–37°C. This has the effect of compressing the waveform in the *Bmal1*-/- example, reducing the apparent slope of a regression line drawn through the temperature waveform prior to mealtime. If the scales are made equivalent, the waveforms look more similar. The body temperature waveforms in these *Bmal1*-/- examples may have more ultradian variation, but body temperature clearly is rising over the 1–4 hours preceding mealtime. Moreover, as discussed in Point 1c above, there are strong indications that the waveform in Fig. S3D (Figs. 3 and 8 here) is misaligned and that temperature rises in anticipation of mealtime at least an hour earlier than this figure suggests. The data therefore appear to contradict the stated claim in the paper.

2e. A critical interpretive issue in any study of food-entrainable rhythms is whether the animals tested can tolerate feeding schedules that limit the amount or duration of food availability. If the subjects cannot tolerate food restriction, due to species characteristics or metabolic effects of lesions or gene manipulations, then attenuation or absence of food anticipatory rhythms in a particular group of animals may be inconclusive. Mice are especially vulnerable to restricted feeding, due to their small size and high metabolic rate. Consequently, it is standard procedure in food restriction studies of mice to gradually, rather than abruptly, reduce the duration of the daily meal over several days. This procedure would seem all the more important for studies of *Bmal1*-/- mice, given that this gene knockout is associated with metabolic deficiencies [27,28] and progressive arthropathy that limits mobility by 3–4 months age [29]. In Fuller et al [12], and in their 'Reply' [30] to a 'Technical Comment' [15], the wording indicates that food was abruptly limited to 4 h/day, without a gradual reduction in meal duration, and was placed on the metal bars of the cage top, requiring the mice to reach up to bite off pieces to eat. A reasonable concern, therefore, is that any attenuation of food anticipatory rhythms in *Bmal1*-/- mice may be secondary to poor health due to inadequate food intake.

These concerns appear to be warranted. Fuller et al report that *Bmal1*-/- mice exhibited '*torpor*', i.e., a severe decline in body temperature at one or more times of day, on one or more days. The authors state explicitly that *Bmal1*-/- mice "*often slept or were in torpor through the window of restricted feeding*, (emphasis ours) *requiring us to arouse them by gentle handling after presentation of the food to avoid their starvation and death during restricted feeding*". Hungry mice (even if asleep) will arouse and orient immediately when the door of their isolation chamber is opened at mealtime to place food in the cage. A mouse that has to be physically handled to be aroused is very likely either sick or profoundly hypothermic (or both). In the original text, the authors do not report body weight or food intake data. In their Reply [30] to the Technical Comment [15], the authors again provide no data, but do state that the heterozygous and null mutant mice ate 85% of ad-libitum intake during the 4 h daily meals, and that the null mutants did not lose weight. This is very puzzling, for at least two reasons. First, even wildtype mice lose body weight when restricted to 85% of ad-libitum intake (relative to their starting weight or to ad-lib fed controls). Second, and more importantly, if *Bmal1*-/- mice did not lose body weight, then why did they exhibit bouts of hypothermia severe enough to prevent them from arousing spontaneously when food was placed in the cage?

There are numerous studies in the literature showing that wildtype mice and rats typically lose weight when food restricted, particularly when the duration of food access is 4 h or less. To evaluate whether *Bmal1*-/- mice are somehow protected from this effect, two laboratories have independently collected food intake and body weight data from wildtype and *Bmal1*-/- mice fed according to the procedure of Fuller et al (4 h/day, food on cage tops). In one lab (W. Nakamura, personal communication, Dec 2008), the null mutants lost significant weight when the food was placed on the cage tops during two days of ad-lib food access (one *Bmal1*-/- mouse had to be taken out of the procedure). Evidently, even without food restriction, *Bmal1*-/- mice have physical limitations that may impair their ability to reach food available in standard cage top food hoppers. Food was therefore placed on the floor for 5 additional baseline days and 4 days of food restriction (4 h/day). A 25% weight loss 'endpoint' criterion was established for termination of the experiment, to prevent death. Three of 5 *Bmal1*-/- mice reached the 25% weight loss criterion on day 3 of restricted feeding and one reached it on day 4. Weight loss in the remaining *Bmal1*-/- mouse was 18% on day 4. Weight loss in 6 wildtype mice averaged 7.5% after one day of restricted feeding, and 9% after 4 days.

**Figure 9:**
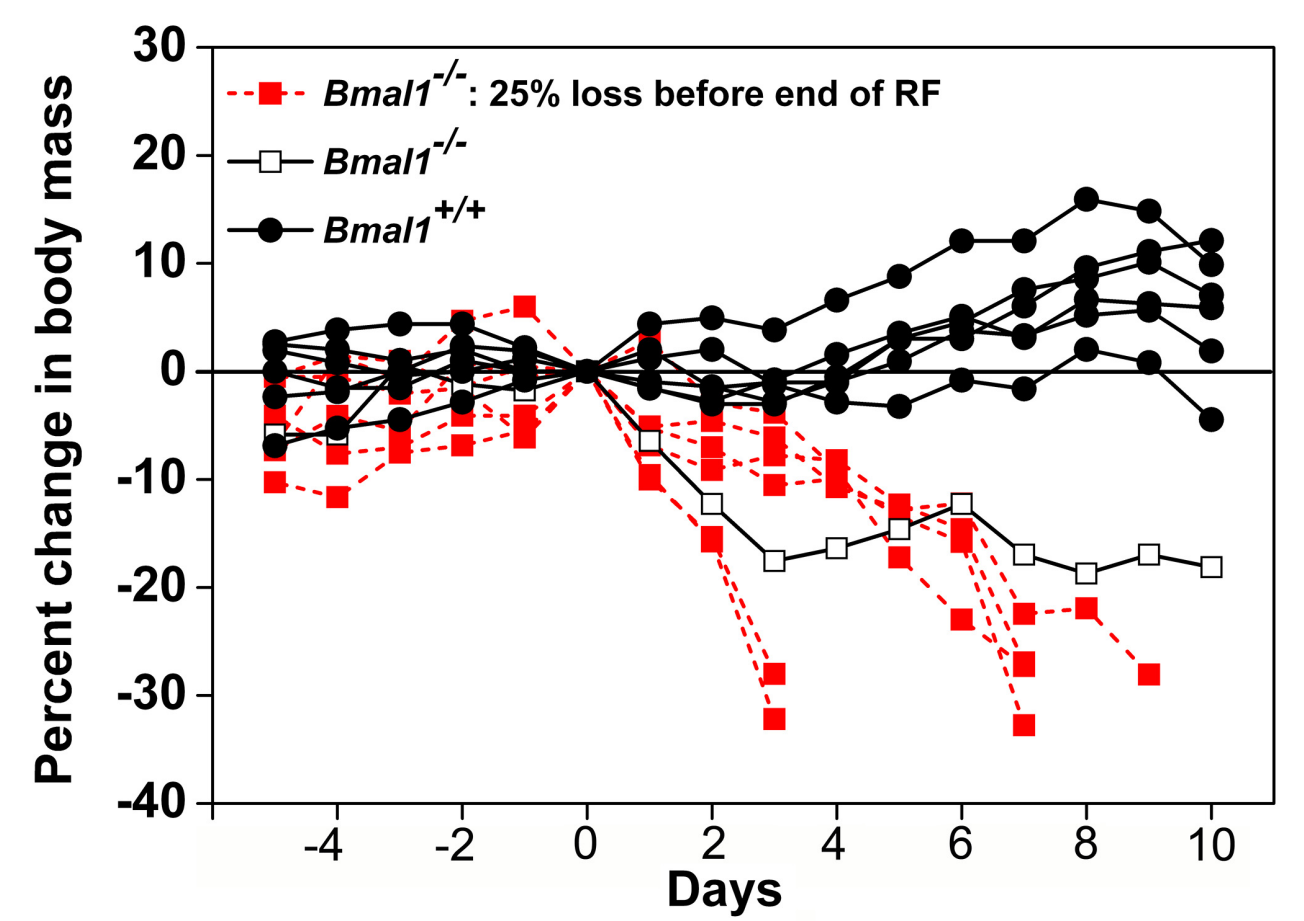
**Percent change of body weight in wildtype and *Bmal1*-/- mice during restricted daily feeding (J. Pendergast and S. Yamazaki, unpublished)**. Body weights of wildtype mice (grey lines, N = 6) and *Bmal1*-/- mice (red and black lines, N = 7) during ad-lib food access (days -5 to 0) and 4-h/day restricted food access (days 1–10), expressed as percent change from day 0. A 25% body weight loss was established as an endpoint criterion, at which time mice were returned to ad-lib food access, to prevent mortality. Only one *Bmal1*-/- mouse (black line) remained above the endpoint criterion over the 10 days of restricted feeding.

In the second lab (J. Pendergast and S. Yamazaki, personal communication, Jan 2009), two sets of wildtype and null mutant mice were tested. The first set consisted of 2 *Bmal1*-/- and 5 wildtype mice, of various ages, with body weights in the 22–28 gm range. These mice were maintained in breeder cages with corn cob bedding and nesting material, in an ambient temperature of 22.5°–25.5°C. Food was placed on the cage tops within 4.5 cm of the floor. When food was abruptly restricted to 4 h/day for 4 days, wildtype mice remained within ± 2% of starting weight, while the two *Bmal1*-/- mice lost 8% and 9% body weight, respectively. The second set consisted of 7 *Bmal1*-/- mice and 6 age-matched (5–8 weeks) wildtype mice. These mice were housed in standard recording cages with locked running wheels, in DD and 22 – 23°C, with food placed on the cage tops as in Fuller et al. Under these conditions, when food was abruptly limited to 4 h/day for 10 days, the *Bmal1*-/- mice lost weight dramatically, all but one reaching the 25% endpoint criterion within 3–9 days (Fig. 9).

Note that in both laboratories, body weights were measured after the daily mealtime, which underestimates weight loss sustained by the mice at meal onset, 20 h after their last meal. The less severe weight loss evident in the first set tested by Pendergast and Yamazaki may be a result of warmer housing conditions (nesting material and higher cage temperature closer to thermoneutral) that would have reduced energy expenditure. Notably, Fuller et al [30] state that cage temperatures in their study were 22 ± .1°C, i.e., below thermoneutral for mice (but see point 3f, below). A third laboratory has also reported rapid weight loss in *Bmal1*-/- mice abruptly restricted to 3 h food/day [18]. In that experiment, ~80% of the *Bmal1*-/- mice died under this feeding protocol, but mortality rates dropped to zero when a gradual food restriction protocol was adopted. Thus, the impact of food restriction schedules on body weight in mice is affected by environmental conditions (e.g., cage temperature below thermoneutral and availability of bedding) and feeding protocol (e.g., abrupt versus gradual reduction of food intake and location of food). Given the methodological details provided by Fuller et al [12,30], the undocumented statement that their *Bmal1*-/- mice did not lose body weight is puzzling.

### 3. Data or methods missing or inconsistent

In this section, we identify data or methodological details that are missing from the manuscript, but that are required to support the major claims, to assess the reliability of the reported effects, or to replicate the experiments.

3a. The total number of mice used, and the numbers per group, are not reported (with one exception; the number of AAV-BMAL1 mice contributing to the preprandial group average temperature in Figure two D is stated as 4 in the figure legend).

3b. The age of the mice at the time of behavioral testing is not reported. Age is a non-trivial methodological detail; as noted, by the age of 14 weeks *Bmal1*-/- mice develop progressive arthropathy and become less mobile, which could attenuate the expression of food anticipatory activity and impair their ability to retrieve food and eat a sufficient amount in 4 h to remain healthy.

3c. The success rates for injection placement, *Bmal1 *rescue and circadian rhythm rescue are not reported. It is technically non-trivial to limit spread of microinjected viral vector to a relatively small target structure (e.g., SCN or DMH). The degree of difficulty is increased when bilateral injections are made (both have to limit expression to the target). To guide replication studies, it would be simple and appropriate to report the total number of mice receiving injections, the number in which *Bmal1 *expression was restored unilaterally, bilaterally or outside of the target area, and the number in each category that exhibited rescue of rhythms.

3d. Figure two D in Fuller et al [12] is a plot of group mean body temperature averaged in 1 h time bins for 4 h before meal onset and 1 h after meal onset, in *Bmal1*+/-, *Bmal*-/- and AAV-BMAL1 mice. This graph shows body temperature rising in anticipation of mealtime in the heterozygous mice and in DMH-rescued AAV-BMAL1 mice but not in the *Bmal1*-/- mice. As noted, the authors state that *Bmal1*-/- mice "*often slept or were in torpor through the window of restricted feeding, requiring us to arouse them by gentle handling after presentation of the food to avoid their starvation and death during restricted feeding*". If some animals were severely hypothermic (< 31°C) at mealtime, it should be made clear in the figure legend as to whether these mice were included in the group averages. If these mice, or certain days, were not included, this should be indicated and the exclusion criteria clearly specified.

3e. In the Supplementary methods (p. 4), Fuller et al state that food restricted *Bmal1*-/- mice tested in LD 12:12 "*may go into torpor during the night and forage more consistently during the day*". However, no rationale or evidence is provided to support this statement. A reasonable assumption is that the incidence of torpor would increase with time since the last meal. Consequently, torpor should be far more likely to occur during the light period just before mealtime, particularly in *Bmal1*-/- mice that are supposed to have no circadian rhythms in LD (e.g., see Fig. 1 in [12]). The same considerations apply to restricted feeding in DD. Given the counterintuitive statement made by Fuller et al with respect to the timing of torpor in *Bmal1*-/- mice housed in LD or DD, it would be appropriate to provide the data supporting these statements.

3f. In the Reply [30] to the Technical Comment [15], Fuller et al state that '*torpor is a normal defense mechanism used by mice when faced with a 20-h fast in a cool laboratory (22°C)*'. In the 'corrected' supplementary materials (Science, Oct 31, 2008), cage temperature is reported as 22 ± .1°C. However, in the previous version of the supplementary materials (Science, May 23, 2008, also available on-line), cage temperature was reported as 24 ± .1°C. It is not clear which version is correct, because the change was made without being noted in the list of Corrections published in the Oct. 31 issue.

### 4. Conceptual issues

The Technical Comment [15] on the Fuller et al paper [12] included original data showing that *Bmal1*-/- mice exhibit robust food anticipatory behavioral rhythms if they are gradually adapted to restricted feeding and if food pellets are placed in the cage rather than on cage tops, where it may be difficult for physically frail *Bmal1*-/- mice to reach. In the Reply [30] to the Comment, Fuller et al make several inaccurate or conceptually untenable statements that may mislead readers less familiar with the methods and literature in this area of research.

4a. In their studies of food anticipatory rhythms, Gooley et al [11] and Fuller et al [12] used intraperitoneal transponders to record general cage activity and body temperature by telemetry. Other laboratories have failed to confirm their findings using overhead passive infrared motion sensors to measure activity either directed at food hoppers or occurring anywhere within the recording cage [13-16]. Fuller et al [30] state that these other studies '*measure food-seeking behaviors rather than circadian rhythms'*. According to Fuller et al, locomotor activity measured by overhead motion sensors is a '*food-seeking behavior' *that reflects homeostatic factors (caloric deprivation and hunger), whereas activity and temperature measured by telemetry are '*unrelated circadian-driven physiological responses*'. Food anticipatory activity rhythms exhibited by *Bmal1*-/- mice in other laboratories [15,17,18] are therefore considered to be the product of a homeostatic, 'hourglass' process (activity driven by hunger and terminated by feeding), rather than a true circadian oscillator, which Fuller et al claim to have disabled by *Bmal1 *knockout, and Gooley et al by DMH-ablation [11]. According to Fuller et al, the failure of other labs to confirm their results is because these labs are '*measuring different things'*.

However, these arguments are neither intuitive nor consistent with available evidence. It is not at all clear how a properly functioning transponder in the intraperitoneal cavity, such as used by Fuller et al, could fail to register activity that is sufficient to trigger an overhead motion sensor, such as used by other labs. Thus, it seems unlikely from the outset that telemetry and overhead motion sensors could be differentially sensitive to homeostatic and circadian factors. In fact, three labs have conducted direct comparisons of the two measures in rats [31] and mice [[16]; M. Sellix and M. Menaker, personal communication, Nov 2008], and in each case, transponders and overhead motion sensors produced virtually identical results. Therefore, the failure of other labs to confirm the results of Fuller et al [12] and Gooley et al [11] is clearly not because these labs are '*measuring different things'*.

Fuller et al's claim that body temperature is also an '*unrelated circadian-driven physiological response' *is similarly unsubstantiated. Fuller et al provide no theoretical rationale for how body temperature could be immune to homeostatic factors (caloric deprivation) and thermogenic effects of eating and ongoing locomotor activity; in fact, their own data indicate that temperature is drastically affected by food restriction (their mice were severely hypothermic). Unsubstantiated claims about the special value of temperature as a circadian endpoint for studies of food anticipation are doubly disconcerting, as these may encourage the unnecessary use of invasive and expensive procedures (intraperitoneal implants and radiotelemetry).

4b. Fuller et al [30], in their Reply to the Technical Comment [15], state that *'the assertions made by Mistlberger et al. concerning the role of the DMH in food-entrainable circadian rhythms are not accurate. We and Mieda et al. did not find that the DMH is one of "a number of" brain regions whose clock gene expression is "synchronized by scheduled feeding." What was found is that the DMH is the only region of the brain that has self-sustained cycles of clock gene expression induced de novo by restricted feeding'*. These comments are remarkable in misrepresenting both the Technical Comment and the results of Mieda et al [32]. Mieda et al showed that the DMH in intact, food restricted wildtype mice exhibits a daily rhythm of *mPer1 *and *mPer2 *gene expression that persists during total food deprivation. These data do not establish that DMH clock gene rhythms are 'self sustained', i.e., generated intrinsically. DMH clock gene rhythms could be driven by inputs from elsewhere in the brain or the periphery, related to sensory, motor, metabolic or other correlates of food anticipatory rhythms in behavior or physiology. There certainly are many other brain regions in which clock gene rhythms are reset by restricted feeding and persist during food deprivation [33-39]. Moreover, Fuller et al's statement that rhythms of *Per *gene expression in the DMH are induced *'de novo' *by daytime feeding is itself not accurate. While this seems to be generally overlooked, Figures 4 and 5 in Mieda et al [32] clearly show that there is daily rhythm of *Per1 *gene expression in the DMH of ad-lib fed mice, with an approximate 3–4 fold increase in expression at ZT13 compared to ZT7. The amplitude of the rhythm only appears low in those figures due to the scaling of the y-axis. During restricted feeding, the phase of the rhythm was shifted and its amplitude increased at least 2-fold. Thus, DMH rhythms are reset and amplified, not induced *de novo*, by daytime restricted feeding schedules. A rhythm of DMH *Per1 *expression in ad-lib fed mice has also been reported by Moriya et al [16], one or two circadian cycles of *Per2 *expression have been observed in the DMH, arcuate nucleus and adjacent anterior hypothalamic areas in mediobasal hypothalamic explants from ad-lib fed PER2:luc mice [36]. Thus, contrary to Fuller et al's reading of the literature, the DMH is no different from many other brain regions that also exhibit daily rhythms of clock gene expression during ad-lib food access that can be reset and amplified by restricted feeding schedules.

## Conclusion

In this review of Fuller et al [12,30] we have identified a large number of flaws in the evidence supporting the claim that a *Bmal1*-dependent circadian mechanism in the DMH is sufficient to drive food-entrainable rhythms of activity and body temperature in mice. While numerous, these flaws are by no means minor. Our overriding objective here is to promote methodological rigor, to enable us to arrive at a clear view of how the brain synchronizes behavior and physiology to daily feeding opportunities. When new, potentially seminal findings are presented, we are obligated to scrutinize the evidence carefully to ensure that it meets standards sufficient to become part of the foundation for future work. The evidence as presented in Fuller et al [12] clearly does not meet the necessary standards.

## Competing interests

The authors declare that they have no competing interests.
